# Castor Oil-Based Polyurethane/S2 Glass/Aramid Hybrid Composites Manufactured by Vacuum Infusion

**DOI:** 10.3390/polym14235150

**Published:** 2022-11-26

**Authors:** Joziel Aparecido da Cruz, Eduardo Fischer Kerche, Otávio Bianchi, Andrea Manes, Sandro Campos Amico

**Affiliations:** 1 PPGE3M, Federal University Rio Grande do Sul (UFRGS), Porto Alegre 91501-97, RS, Brazil; 2 Politecnico di Milano, Department of Mechanical Engineering, 20156 Milan, Italy

**Keywords:** aramid, glass, hybrid laminates, castor oil polyurethane, vacuum infusion, energy absorption, dynamical mechanical response

## Abstract

This study evaluates the hybridization effect of S2-glass/aramid on polyurethane (PU) composites produced by vacuum infusion. Different laminates were produced with similar thickness (around 2.5 mm), using, as reinforcement, only aramid fabrics (five layers, named as K_5_) or only S2-glass fabrics (eight layers, named as G_8_). Furthermore, hybridization was obtained by manufacturing symmetrical hybrid inter-ply laminates, with four S2-glass layers and two of aramid, (G_2_K)_S_ and (KG_2_)_S_. The mechanical response of the laminates was evaluated in tensile, interlaminar shear strength, dynamical mechanical analysis and quasi-static indentation tests, and related to their morphological characteristics. The main results show that the pure glass composites presented less voids, but a higher density as well as higher tensile stiffness and strength. The aramid laminates showed a high capability for absorbing impact energy (ca. 30% higher than the pure glass laminates), and the hybrid laminates had intermediate properties. More importantly, this work shows the possibility of using a polyurethane matrix for vacuum infusion processing, effective even for aramid/S2-glass hybrid composites with thermoset polyurethane resin. This study is therefore promising for impact absorption in applications such as protective armor. The studied hybrid laminate may display a suitable set of properties and greater energy absorption capability and penetration resistance for impact applications.

## 1. Introduction

Continuous-fiber polymer composites have been used in many fields, such as aeronautical, naval, civil, and also military applications [1]. For more demanding applications with high-performance components, pre-impregnated fabrics (or prepregs) are commonly used as raw-materials [2]. Laminates with high fiber content are obtained when prepregs are employed, which decreases the overall component weight and improves the specific mechanical response. Despite the well-known benefits of prepregs, these materials are expensive and not widely available. Moreover, non-environmentally friendly polymers are used (e.g., epoxy) for prepregs [3].

In addition, conventional thermoset resins are commonly stiff and brittle, which may present a problem when the composite is destined for high impact response applications [4,5]. Alternatively, elastomeric polymer matrices have been used in shock absorbers, impact resistance panels, and other engineering applications, due to their high flexibility and damping properties [4]. When a projectile impacts the fabric, primary yarns engage the projectile and absorb most of the kinetic energy. Transverse deflection of the main yarns then pulls secondary yarns that are not in direct contact with the projectile. These yarns assist in the dissipation of load and increase the overall component energy absorption [4].

Elastomeric matrices can improve energy absorption in impact. Khodadadi et al. [4] investigated the high-speed impact response of neat Kevlar fabric, stiff Kevlar/epoxy composite and a soft Kevlar/rubber composite, and showed that the Kevlar fabric and soft Kevlar/rubber composite absorbed about 200% and 400% more energy than the stiff Kevlar/epoxy, respectively. Gopinath and coworkers [6] suggested that Kevlar composites with soft matrices have better impact resistance than Kevlar composites with rigid matrices because the Kevlar yarns are less constrained by the soft matrix during impact and are able to dissipate more impact energy. On the hand, for a stiff composite, only yarns close to the impact zone contribute to energy dissipation during impact [7].

Polyurethanes (PUs), in particular, may display thermoplastic, elastomeric, or thermoset behavior, depending on the chemical architecture of their monomers [8]. Due to the possibility of using chemicals, such as chain extenders, surfactants, and catalysts, PUs are highly versatile, and their use and diffusion are increasing in several fields, such as the automotive, construction, medical, electrical fields, among others [9,10,11]. PUs are commonly produced by the reaction of petroleum-based molecules containing hydroxyl (-OH) groups with isocyanates (-NCO) [12]. However, vegetable oils can also be used as raw material to produce bio-based PUs, with advantages such as low toxicity, inherent biodegradability, and high purity.

Considering green chemistry principles and environmental concerns, vegetable oils occupy a very important position as renewable resources to develop new valuable products [12,13]. Castor oil (CO) is a natural polyol with many applications in the polymer industry, particularly for PUs. CO has attracted great attention owing to its availability, cost-effectiveness, biodegradability, eco-friendliness, and ease of extraction from castor seeds [12]. Furthermore, the blending of petroleum-based oils with bio-based precursors enables the tuning of properties such as reactivity, viscosity and reaction kinetics, increasing its application range [14,15].

Many techniques have been used for composites manufacturing [3,5,16]. Vacuum Infusion (VI), in particular, enables the production of composites with high V_f_, low void content, and high surface quality [1,5]. Moreover, VI has low-cost, is readily available and does not demand robust equipment and molds. The use of PU resin for VI processing, however, poses challenges related to maintaining a relatively constant and low viscosity for a long processing time; also the PU system is moisture sensitive [12]. In a previous study by our group [15], an elastomeric PU resin formulation adequate for VI processing was developed using a blend of polyols (polyester-based vegetable oil and petroleum-based polyether oil). The resulting PU combined low-viscosity, suitable reactivity time and hydrolytic resistance of the former polyol with the hydrophobic character and the sustainable aspect of the polyol from castor oil, overcoming some limitations and allowing the use of a polyurethane resin in VI process [17,18].

Mohamed et al. [19] reported on the manufacturing and mechanical response of laminate composites produced by VI. Two types of commercial rigid PU were used as the matrix, along with plain-weave E-glass fabrics as reinforcements. The impact absorption energy of the components was high compared to structural composites produced by VI in the literature with conventional thermoset polymers, such as polyester and vinyl ester resins. Furthermore, no volatile organic components were released when the PU matrix was used, representing another environmental advantage [17,20]. Indeed, Dai, X. et al. [21] studied different techniques for manufacturing E-glass fiber-reinforced PU composites and reported that the composites produced by VI presented better mechanical performance (bigger tensile elastic modulus 30%) than those by hand lay-up or compression molding, mainly related to a more homogeneous micro-structure.

Focusing now on reinforcement, aramid fibers (AF) are widely used in composites for high-impact energy absorption, presenting high protection against fragmentation and ballistic threats with reduced weight and thickness [1]. However, the infiltration of AF using liquid molding techniques is difficult due to their low wettability compared to glass fibers [5,22]. In this context, hybridization of these fibers can facilitate processing, since infiltration is enhanced, and resin distribution becomes more even. Silva et al. (2020), studied Kevlar 49/R-glass hybrid VI molded laminates and concluded that inclusion of R-glass fabrics increased permeability (60% higher) compared to pure aramid, and a synergistic effect of hybridization was identified [5]. In this study the hybridization in laminates affected their mechanical response (50% higher in tensile modulus), enabling a wider range of mechanical properties that could not be achieved with a single fiber type, including increased energy absorption and reduced indenter penetration under impact [1,23,24].

Different tests can be used to evaluate the energy absorption capacity and impact resistance of composites, and several researchers [25,26,27] have shown similar trends comparing low-velocity impact and quasi-static indentation (QSI) tests. Dynamic mechanical analysis (DMA) is another technique whose results are commonly associated with energy dissipation under impact [23]. Reddy et al. [28] found that laminates with lower short-beam strength (SBS) showed higher energy absorption and ballistic limits due to dissipation of energy in lateral directions [28]. Yudhanto et al. [29] evaluated continuous glass-fiber reinforced polymeric composites and showed that higher matrix ductility inhibits the growth of ply cracking along the fiber, controlling delamination.

There are just a few papers on the use of PU for the manufacturing of composites by VI, and hardly any on hybrid composites. So, the main goal of this work was to obtain, by the VI process, interply hybrid composite laminates with S2-glass and aramid and a polyol blend (vegetable oil and poly(propylene oxide)) PU resin synthesized in previous work. The composites were characterized to correlate their mechanical response and energy dissipation ability with their morphological and viscoelastic characteristics.

## 2. Materials and Methods

### 2.1. Materials and Laminate Manufacturing

**Figure 1:**
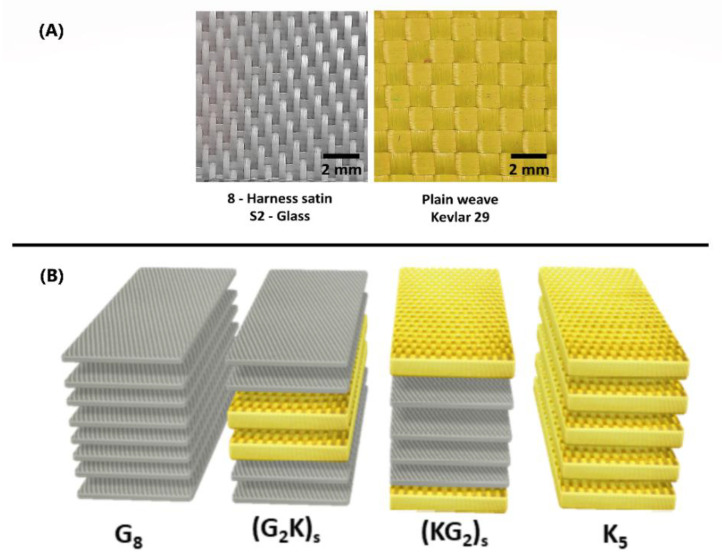
S2-glass and aramid (Kevlar 29^®^) fabrics (**A**), stacking sequence and nomenclature adopted for the laminates (yellow for Kevlar 29^®^, grey for S2-glass) (**B**).

Plain-weave Kevlar 29^®^ (440 g/m^2^, 0.62 mm, 7 threads/cm, ρ = 1.45 g/cm^3^) fabrics from Dupont, and 8-harness satin S2-glass (302 g/m^2^, 0.24 mm, 22 threads/cm, ρ = 2.49 g/cm^3^) fabrics from Hexcel, shown in Figure 1A, were used as reinforcements,. The composite matrix used in this study was a elastomeric polyurethane—PU (ρ = 1.05 g/cm^3^) composed of a polyol blend (vegetable oil and poly(propylene oxide)), reported in details in a previous work [15]. Figure 1B illustrates the four types of laminates produced.

Two of them have a single fiber, Kevlar 29^®^ (K_5_) and S2-glass (G_8_), and two are symmetric hybrids, namely (KG_2_)_S_ and (G_2_K)_S_. The stacking sequences and number of layers were chosen to obtain a similar final thickness of ~2.5 mm for all configurations. To evaluate fabric compaction, all fabric stackings were tested under compression in a universal testing machine (Instron brand, model 3382, Norwood/MA/USA), with circular plates [22]. The compression load (or pressure), the distance between plates, the VI cavity height and, therefore, the expected fiber volume fraction (V_f_), were correlated. Each stack of fabrics was placed on plates positioned 5 mm apart, and the loads required to bring the reinforcement to lower thicknesses were recorded.

Prior to composite manufacturing, the fabric layers were dried in an oven for 12 h at 120 °C. Since the polyurethane is sensitive to moisture (i.e., hydrophilic), drying is critical, since any active hydrogen, such as that in the water, can react with the isocyanate group producing CO_2_ gas, which expands and generates air bubbles (increasing the void content), and decreasing the final quality of the composite material [15,17].

**Figure 2:**
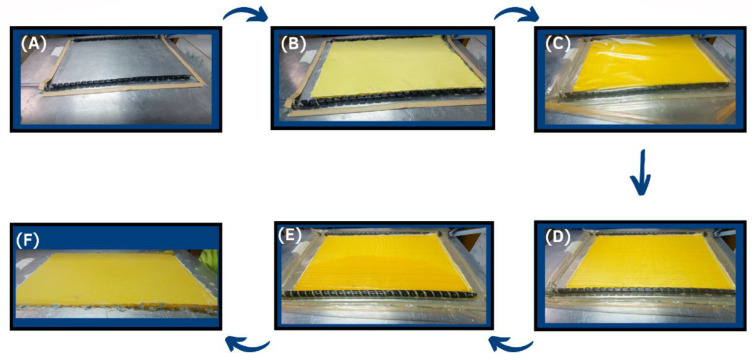
Manufacturing steps for VI processing: (**A**) surface preparation, (**B**) fabric stacking, (**C**) vacuum bag closing, (**D**) closed system, (**E**) resin permeation, and (**F**) final laminate.

The system was sealed using tacky tape (Figure 2C) and the cavity was evacuated, removing air and compacting the reinforcement (Figure 2D). The PU resin entered the cavity through an inlet under the imposed vacuum (100 kPa = 1 bar), wetting the layers as shown in Figure 2E. The plates were left to cure for 24 h under vacuum, and the laminate was extracted (Figure 2F). The composite was then subjected to post-curing (8 h at 70 °C) until complete PU polymerization. Specimens for testing were later obtained by water-jet cutting.

### 2.2. Laminate Characterization

A polarized light optical microscope coupled with the Carl Zeiss axio Lab A1 image analyzer was used to obtain micrographs of the longitudinal cross-section of the laminates. The laminate density was evaluated following ASTM D792 standard. Their constituent characteristics, namely, overall fiber volume content (V_f_) as well as aramid (V_K_), glass (V_G_), and PU matrix (V_m_) content, were obtained based on ASTM D3171.

Tensile tests were performed according to ASTM D3039, using six specimens (dimensions: 250 mm × 25 mm) for each configuration, which were tested until failure at a crosshead speed of 2 mm/min. Longitudinal strain was obtained with a video extensometer. Short-beam tests were performed according to ASTM D2344 at 1 mm/min, using a span:thickness ratio of 4:1 and for ten specimens (24 mm × 8 mm) for each laminate. These mechanical tests were performed in an Instron universal test machine with 5 kN load-cell.

Dynamic mechanical analysis (DMA) was carried out on cured PU (open casting molded under –1 bar vacuum) and post-cured laminates according to the ASTM D7028 standard, using 60 mm × 12 mm specimens. DMA was performed with a TA instrument, model Q850, using a dual-cantilever clamp at a strain amplitude of 0.1%, frequency of 10 Hz, and heating rate of 3 °C/min from −60 °C to 120 °C. Storage modulus (E′), loss modulus (E”), tan delta (tan δ), and glass transition temperature were determined.

In addition, the effectiveness of the reinforcement, represented by the C coefficient, was calculated from Equation (1) [23].

(1) $$C=\frac{{{(E}_{g}^{\prime }/E_{r}^{\prime })}_{\mathrm{composite}}}{{{(E}_{g}^{\prime }/E_{r}^{\prime })}_{\mathrm{PU}}}$$

where E′_g_ and E′_r_ are the storage modulus values in the glassy (set to −40 °C) and rubbery (set to 40 °C) states, respectively. The reinforcement efficiency was also assessed in terms of the so-called adhesion factor (A), calculated using Equation (2) [23,30].

(2) $$A=\frac{1}{{1\;-\;V}_{f}}\frac{{\tan\;\delta }_{\mathrm{composite}}}{{\tan\;\delta }_{\mathrm{PU}}}$$

where V_f_ is the fiber volume fraction (Table 1), and tan δ_composite_ and tan δ_PU_ are the relative damping values for the composite and polymer matrix (PU), respectively. The higher the A factor, the lower the fiber/matrix adhesion [31].

Quasi-static indentation tests (QSI) were performed according to ASTM D6264 in the same testing machine but with a 100 kN load-cell. Three samples (150 mm × 150 mm) were tested for each configuration. The samples were constrained between steel plates with a circular cutout (ø = 125 mm) and subjected to a concentrated loading in the out-of-plane direction using a hemispherical indenter (ø = 12.7 mm) at the center of the sample. The test was carried out until 18 mm of displacement, followed by full unloading, both at 1.25 mm/min speed.

The obtained QSI, tensile and short-beam properties were submitted to normality and homogeneity of variances tests before applying single variance analyses (ANOVA). When the null hypothesis was rejected, average tests followed using Fisher’s LSD method with 5% significance. Distinct uppercase letters (A, B, C and D) associated with each parameter value represent significant differences between groups.

## 3. Results and Discussion

### 3.1. General Characterization

Table 1 shows the constituents content of the produced laminates. K_5_ presented the greatest overall V_f_ (59.42 ± 0.45%), which was reduced when more layers of S2-glass were used, reaching a minimum for the G_8_ laminate (52.71 ± 1.01%). Da Silva et al. [5] reported the same decreasing trend when glass fabrics were added to the VI laminate. These results are related to the different fabric architecture and the higher aramid compaction for the maximum VI pressure (−1 bar) available during molding. 

As the number of glass layers increases, the void content (V_v_) is reduced, probably due to the higher permeability and better wettability of the S2-glass fabric, facilitating resin flow within the preform [1,5].

Just a few studies in the literature have focused on polyurethane resin for the VI process. Mohamed et al. [17], obtained glass-reinforced PU composites by VI and found a fiber volume fraction around 53% and void fraction around 1%. The fiber content was similar to that of the current work; however, there was a large difference in void fraction (around 5.9% in Table 1) possibly related to the different nature of the PU used. Indeed, compared to the commercial petroleum-based PU formulation, the used vegetable oil (polyester polyol) was more sensitive to moisture, which can affect the reaction and produce more voids on the composites [32].

**Figure 3:**
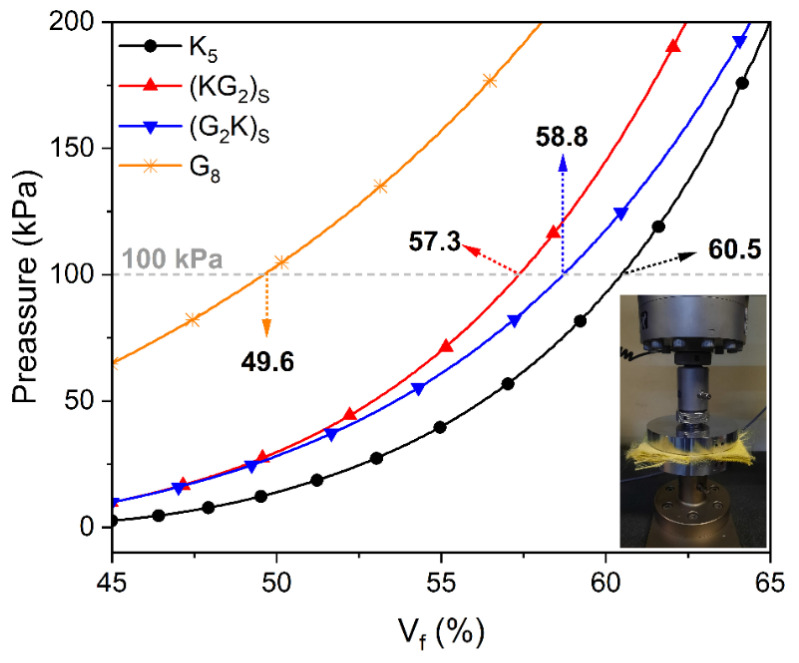
Compression test results for the K_5_, (KG_2_)_S_, (G_2_K)_S_ and G_8_ fabric stackings.

The results concerning constituent content can be better appreciated by the results of the fabric compression test shown in Figure 3. At 100 kPa (≈1.0 bar pressure), the condition experienced by the fabrics in the VI process, the expected fiber content was ≅60% for the aramid fabric and 50% for the S2-glass. The highlighted points in Figure 3, i.e., the expected %V_f_ for each stacking sequence at 100 kPa, agree well with the experimental V_f_ values (see Table 1), including the intermediate results for the hybrid laminates. Indeed, when only S2-glass fiber fabrics were used, greater compaction was obtained (see Figure 3) compared to the other stackings, reaching a higher V_f_.

Laminate thickness and density are also shown in Table 1. Since S2-glass fiber has higher density compared to aramid (and also PU), there is an increase in laminate density when more glass layers are used. Silva et al. [1,23] used the same reinforcements, but with epoxy resin (widely used in VI process), and reported higher density (≈10–20%) compared to the current study for similar laminates, resulting from the lower density of the polyurethane resin [15]. Overall, the results in Table 1 (constituents content, density and thickness) show intermediate values for the hybrid laminates, as reported in the literature [1,23].

**Figure 4:**
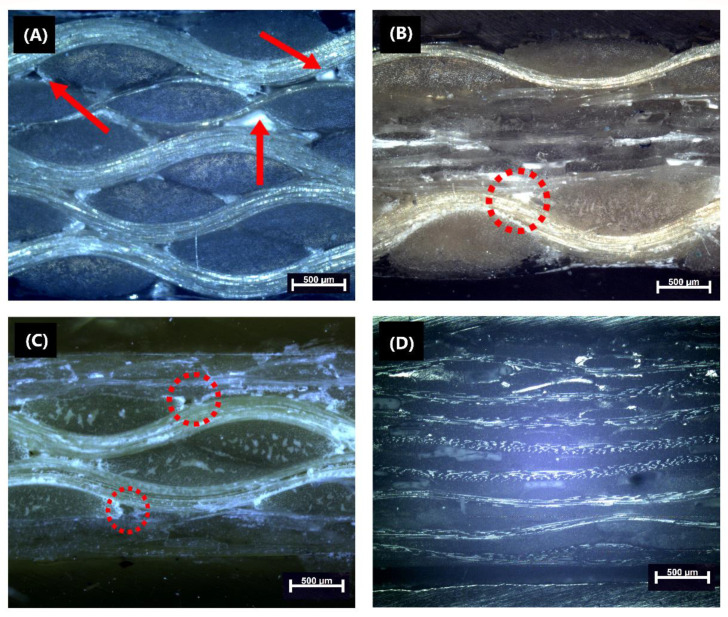
Cross-section micrographs of: (**A**) K_5_; (**B**) (KG_2_)_S_; (**C**) (G_2_K)_S_ and (**D**) G_8_ (50×).

Figure 4 displays micrographs of the longitudinal cross-section of the composites. Some inter-bundle and resin-rich areas are present in the hybrid laminates, indicated by circles in Figure 4B,C. The low compatibility of fabrics, due to the different architecture and entanglement of bundles affects resin impregnation, results in less homogeneous distribution for the hybrid laminates compared with the K_5_ and G_8_ composites (Figure 4B,C).

Voids can be seen, highlighted by red arrows in Figure 4A, mainly in the K_5_ laminate, due to the high void content (see Table 1). Resin-rich regions are less evident because aramid can allow greater compaction at the pressure (−1 bar, see Figure 3) available during the vacuum infusion processing [1].

Moreover, it is possible to observe a greater content of voids, especially near the aramid bundles in Figure 2A (indicated by the arrows) which were reduced by using more glass layers (Table 1). The higher crimp and higher number of longitudinal yarns of aramid fabrics compared to S2-glass hinders resin flow, decreasing in-plane permeability and leading to a poorer resin distribution [5].

### 3.2. Mechanical Properties of the Laminates

**Figure 5:**
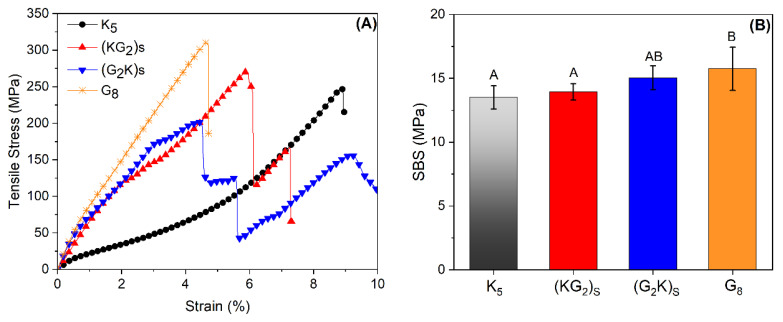
Typical tensile stress-strain curves (**A**) and short beam results for samples (**B**).

Figure 5 shows typical tensile stress-strain curves for all laminates. An abrupt load drop was seen when pure glass and pure aramid laminates reached the maximum tensile load. On the other hand, failure of the (G_2_K)_S_ and (KG_2_)_S_ showed first the failure of the middle glass layers (due to its lower ultimate strain) at 203 and 276 MPa, respectively. After that, stress increased again until the aramid layers failed at 123 and 169 MPa, respectively. The (G_2_K)_S_ behavior was more similar to that of G_5_, compared to the (KG_2_)_S_, even though the only difference was the layer stacking, i.e., in the latter, aramid was at the outer layers.

Table 2 compiles the experimental TS, Et and ɛfirst failure for all composites. For the G8 composite, tensile strength was 315.8 MPa, modulus of elasticity 19.8 GPa and maximum strain (first failure) 4.1%. Mohamed et al. [17] studied E-glass/polyurethane composites, equivalent to the G8 composite of this study, and reported somewhat close values for TS and Et, at 309 MPa and 21.4 GPa, respectively. With increase in the number of glass layers, there was an increase in Et and a decrease in maximum strain. As for TS, when a single central layer of S2-glass was used, there was a reduction in TS compared to pure aramid (K_5_). The first layer that ruptured was the S2-glass (275 MPa), and the remaining four layers of aramid were not sufficient to overcome the TS of the K_5_ composite. There were statistically significant difference among the TS values of K_5_, (K_2_G)_S_ and (G_2_K)_S_ (according to ANOVA with p factor > 0.05) as showed in Table 2.

Similar behavior was reported for the aramid/S2-glass hybrid [23] i.e., the glass fiber layers failed first due to the lower maximum strain. With a stress drop, however the aramid layers were able to support the load until the ultimate load was reached. Comparing the TS of hybrids and pure laminates, the hybridization (G_2_K)_S_ promoted the improvement of 56% in stiffness and 10% in strength when glass fiber was added. These increases in tensile properties were not greater, since the transfer of load from the S2-glass layers to the aramid outer layers was not efficient, with the composite failing completely when the S2-glass central layers failed.

Regarding the short-beam strength (SBS) results compiled in Table 2, the highest values are seen for G_8_, the lowest for K_5_, and the hybrids have intermediate values. There were significant differences between K_5_ and G_8_. SBS is governed by the matrix and the interfacial strength between fiber and matrix [28], due to the better compatibility between PU and glass fibers compared to aramid fibers and by the reduction in void content (see Table 1 and Figure 4A–D), both contributing to its higher SBS value compared to the K_5_ laminate. Similar results for aramid, glass and hybrid laminates can be found in the literature [5,28,33].

### 3.3. Laminate Viscoelastic Response

**Figure 6:**
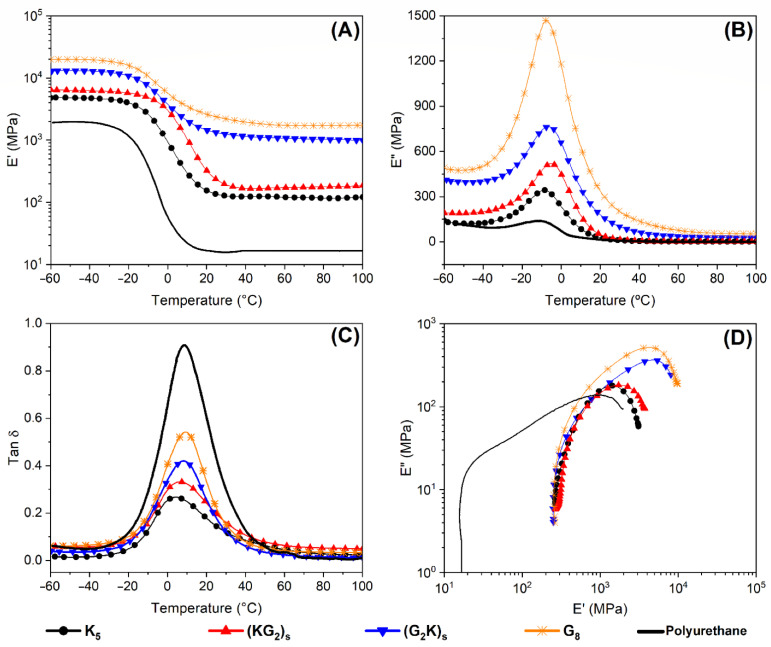
DMA results for PU and laminates: (**A**) storage modulus; (**B**) loss modulus, (**C**) tan δ, and (**D**) Cole-Cole curves.

Figure 6 shows curves from the dynamical mechanical analysis of the laminates and neat PU resin. Storage modulus, loss modulus, and tan δ as a function of the temperature are given in Figure 6A–C, and Figure 6D shows the associated Cole-Cole plots. The main results are also compiled in Table 3. For all samples, E′ decreased with the temperature. In the glassy region (−20–20 °C), the storage modulus values gradually decreased, while in the rubbery plateau (−20–20 °C), there were no significant changes.

The G_8_ laminate had a greater storage modulus, which decreased when aramid fibers were incorporated, as seen for the tensile modulus. A considerable increase in storage modulus, especially in the polymer rubbery region (−20–20 °C), was seen for the laminates in comparison with the pure PU due to the reinforcing effect from the fibers [31].

The polymer in the glassy region had low mobility (vitreo stage), characteristic of pseudo-solid material, i.e., storage modulus greater than loss modulus [15,34]. The polymer macromolecule and the reinforcement are close and tightly packed, resulting in high storage modulus. As the temperature increased, mobility of the components increased, losing their packed configuration and acquiring a pseudo-liquid behavior, i.e., loss modulus higher than the storage modulus.

No significant difference was noticed in T_g_ among composites and polyurethane. A considerable increase in storage modulus, especially in the rubbery polymer region (−20–20 °C), was seen for the laminates compared to the elastomeric neat PU. This is related to the reinforcing effect promoted by the long fibers with a more significant difference above T_g_. Although the used PU matrix consists of a blend of polyols, the tan δ peak for the pure polymer has a single T_g_ probably due to the scale of the DMA investigation, as discussed by Cruz el al. [15].

Thus, the fibers increased the PU matrix capability to resist to deformation, as expected, with a recoverable response. The laminate with the highest E′ was G_8_, with only S2-glass fibers, likely due to the higher stiffness of the glass fiber [20] and the stronger fiber/matrix interaction between the constituents compared to aramid fiber [8,15].

The storage modulus of the hybrid laminates followed the same trend for tensile modulus, even though the samples were subjected to bending loading (dual cantilever clamp) in the DMA [34,35], and the hybrids with S2-glass fibers on the surfaces (G_2_K)_S_ had the highest values. That is, stiffness of the aramid laminate increased with hybridization with S2-glass.

Regarding the C parameter, pure glass laminate showed the lowest value, i.e., 0.08, indicating a less effective reinforcement, perhaps due to the lower V_f_ of that composite, and smaller interfacial area. For laminates with aramid layers, V_f_ was higher, and the C parameter increased, indicating greater reinforcement effectiveness. In other words, as the temperature increased, the laminates with lower fiber content, even for the stiffer fiber (i.e., glass), displayed a more significant drop in E′ at the glass transition, indicating a more pronounced change in mobility of the PU chains for that lower fiber content.

Loss modulus (E″) followed the same trend as E′, and the lower V_f_ of pure S2 glass composites shifted the peak of E″ to lower temperatures. The stronger bonding between S2-glass and PU promoted a higher loss of energy through the interface, increasing the E″ values [35].

The stacking sequence showed a significant effect on these properties. The laminate with aramid fibers on the outer layers, (KG_2_)_S_, presented lower E′ and E″ compared to the other hybrid (G_2_K)_S_. The lower stress transferred on the surface of the (KG_2_)_S_ may cause lower values for both E′ and E″, as reported in the literature [23].

Finally, the highest A value was obtained for the (G_2_K)_S_ laminate, indicating poorer overall interfacial adhesion, thus, greater energy dissipation would be expected [23]. On the other hand, the lowest A value was found for (KG_2_)_S_, related to the aramid positioning on the laminate’s surface, compacting the glass fabrics in the composite interior, improving adhesion between matrix and fabrics, as presented in the MO images (see Figure 4).

Cole-Cole plots have been associated in the literature with the impregnation characteristics of composites [23,34]. In Figure 6D, the sample with only glass fibers presented the lowest semicircle diameter, suggesting a more homogeneous system, as reported in the literature, since the architecture of the used glass fabric (8-hardness satin) facilitates permeation of the fibers compared to the aramid fabric [1,5]. The hybrid laminates again showed intermediate behavior compared to pure aramid and pure glass laminates.

### 3.4. QSI Results

**Figure 7:**
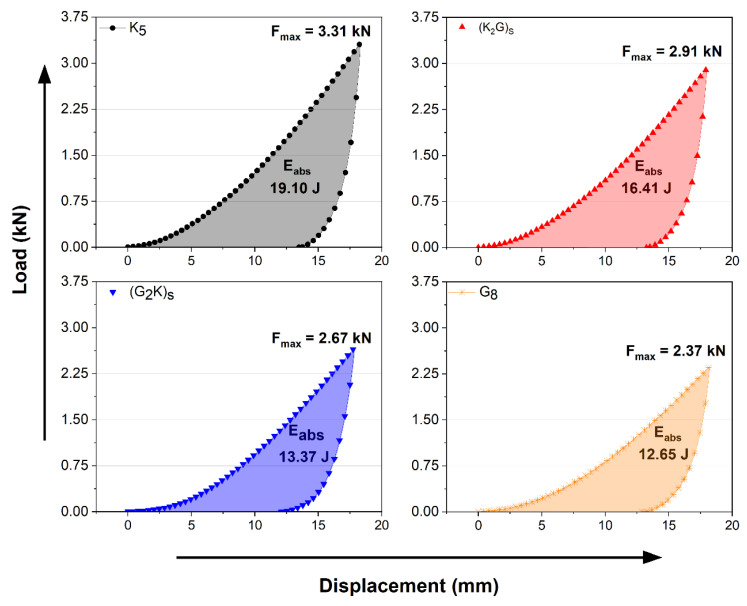
Load/unload QSI results for the laminates.

Figure 7 shows the QSI curves of the studied laminates. The maximum force obtained during the test is shown near the peak of each curve, and energy absorption was estimated from the area of the curve when the laminates were subjected to a displacement of 18.0 mm and then fully unloaded. 

The curves presented in this study differ from those that used conventional thermoset polymers, such as epoxy [23,25]. QSI tests of composites with a stiff matrix show three distinct stages, related to: (i) matrix failure,; (ii) start of fiber failure, and (iii) friction between fibers that can reach complete perforation [23]. However, composites that are more ductile due to the elastomeric polymer can withstand greater deformation until matrix failure starts [36,37], as can be seen in Figure 7.

In QSI tests, Silva et al. [23] showed that the first layers of the laminate are compressed during penetration. Thus, compressive and tensile strengths of the outer layers are important. S2-glass fibers exhibit higher tensile and compressive modulus than aramid (as previously shown in the tensile and SBS tests), and their presence in the first layers seems beneficial. In the second stage, the fibers are sheared and stretched. In the last stage, once the neutral layers are penetrated, the fibers are stretched, justifying the use of fibers with higher strain at break and strength.

Deformation capacity may allow additional damage mechanisms, such as delamination, justifying the use of fibers with higher strain at break and strength, i.e., aramid fibers [23]. This behavior is also related to the high ductility and capacity of energy absorption of PU itself, which hinders the initiation of fiber failure (related to the second stage of QSI test), causing friction and perforation.

Bulut and Erkliğ [25] evaluated S-glass/aramid/epoxy laminates using QSI tests, and the hybrids presented intermediate values compared to pure aramid or S-glass laminates (no positive hybrid effect was noticed), although the hybrid with S-glass fiber at the indentation surface presented the highest maximum force, similar to the current work. The studied hybridization may bring the additional benefit of extending the composite life, since S2-glass fibers on the outer surface are expected to shelter the aramid fibers from deleterious ultraviolet light and water contact. Moreover, the use of the resin used in this study (polyurethane based on a blend of polyester and polyether polyol) is extremely promising, since the properties of this resin combine the hydrophobicity of castor oil and the hydrolysis resistance of the polyol polymer, as reported by Cruz et al. [15]. An initial linear drop was observed after the maximum load for all samples, when the load progressed towards zero in Figure 7. 

**Figure 8:**
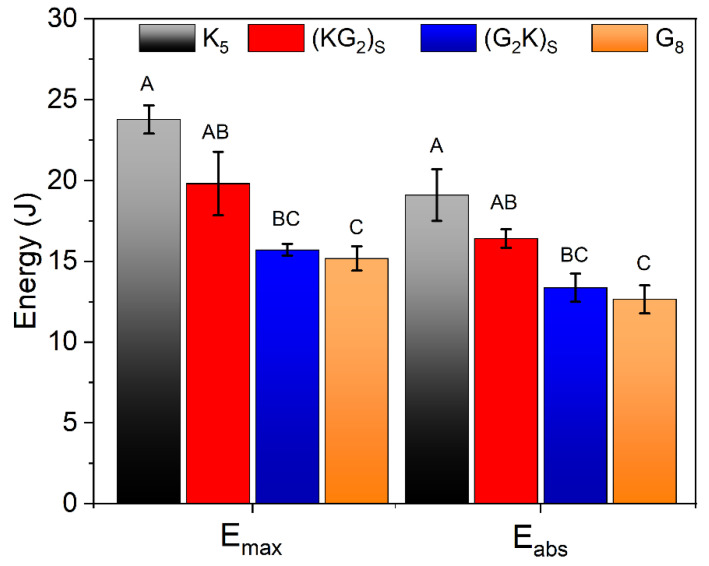
Maximum and absorption energy obtained in the QSI test.

Figure 8 compiles the results of maximum and absorption energy for each laminate. The A, B and C letters represent different families (for each column) according to ANOVA (*p* < 0.05). Regarding, Eabs and Emax, greater energy absorption was seen for the composites with pure aramid due to the higher fiber ductility. Finally, for the hybrid composites, the aramid on the indentation surface was responsible for higher energy absorption. For these composites (i.e., (KG_2_)_S_), ductility of aramid and stiffness of S2-glass make these composites attractive alternatives when a balance between properties is required.

It was interesting to see that aramid hybridization can improve energy absorption. This behavior is inversely related to the tensile and SBS results, given that, as the glass fiber is removed from the faces, there is a decay in these properties since the presence of aramid fiber (more ductile nature) tends to absorb more energy under indentation, as reported by Joana et al. [36,38] who studied carbon, aramid and linen hybrid laminates using a thermoplastic TPU matrix via hot compression and found similar behavior. As the matrix presents elastomeric behavior, it is not expected to show matrix failure (different from stiff matrices), which better exploits the fiber properties, as can be seen in the QSI results.

## 4. Conclusions

In this study, vacuum infusion was successfully used as a manufacturing technique for producing composites using a polyol blend (vegetable oil and poly(propylene oxide)) polyurethane resin developed in previous work by the group. Four laminates were produced, namely pure aramid (K_5_), pure glass (G_8_), and two symmetric interply hybrids (KG_2_)_S_ and (G_2_K)_S_, of similar final thickness (~2.5 mm). Mechanical properties, and viscoelastic characteristics were assessed, and morphological analysis were carried. Based on the results, the following conclusions can be drawn.

The K_5_ laminate presented the greatest overall V_f_ (59.4%), which was reduced when more layers of S2-glass were used, reaching a minimum for the G_8_ laminate (52.7%) due to the greater aramid fabric compaction as indicated by the fabric compression tests. In addition, the void content was higher for pure aramid laminates (11.1%) compared to S2-glass (5.9%) attributed to the difference in fabric architecture (plain-weave and 8-harness satin, respectively). The hybrid stacks showed intermediate behavior to pure laminates.

The tensile strength showed an abrupt load drop when pure glass and pure aramid laminates reached the maximum load, namely 315.8 and 251.9 MPa, respectively. On the other hand, the (KG_2_)_S_ and (G_2_K)_S_ hybrids showed first the failure of the S2-glass layers (due to its lower ultimate strain) followed by an increase in stress until the aramid layers failed at 123 and 169 MPa, respectively. Similar behavior was identified for SBS, i.e., the highest value for G_8_, the lowest for K_5_, and the hybrids with intermediate values, explained by the better compatibility between PU/glass fibers compared to PU/aramid fibers, and by the reduction in void content.

The T_g_ of the PU used in this work was 8.6 °C and no significant differences were noticed in T_g_ among the composites and polyurethane. The low T_g_ of the resin used was responsible for the material displaying elastomeric behavior at room temperature, making it more effective in absorbing impact energy. Furthermore, the stiffer pure S2-glass composites presented lower energy absorption capacities among all samples in the QSI test. Greater energy absorption was seen for the composites with pure aramid due to the greater fiber ductility. The hybridization produced a beneficial effect, combining high reinforcement efficiency with good PU/fibers adhesion.

Finally, the used elastomeric PU matrix prevented premature matrix failure and promoted energy absorption compared with more usual thermoset polymers for composites, also bringing environmental benefits due to the use of a bio-based polyol for the PU synthesis. In all, the material combination studied in this work displays a synergistic effect in the hybrid laminates, e.g., higher energy absorption capacity and puncture resistance, and is very promising for protective applications.

## Figures and Tables

**Table 1:** General characteristics of the laminates.

Laminate	V_v_ [%]	V_K_ [%]	V_G_ [%]	V_f_ [%]	Density [g/cm^3^]	Thickness [mm]
K_5_	11.1 ± 0.2	59.4 ± 1.0	--	59.4 ± 0.4	1.05 ± 0.03	3.01 ± 0.03
(KG_2_)_S_	8.1 ± 0.1	32.0 ± 0.3	25.4 ± 0.2	57.4 ± 0.6	1.13 ± 0.02	2.28 ± 0.18
(G_2_K)_S_	7.3 ± 0.1	33.5 ± 0.1	24.6 ± 0.2	58.1 ± 1.5	1.21 ± 0.04	2.39 ± 0.03
G_8_	5.9 ± 0.1	--	52.7 ± 0.6	52.7 ± 1.0	1.57 ± 0.09	2.41 ± 0.11

Where, overall fibre content (V_f_), and the aramid (V_K_), S2-glass (V_G_) and void (V_v_) volume.

**Table 2:** Mechanical properties of the laminates.

Sample	E_t_ [GPa]	TS [MPa]	ε_first failure_ [%]	SBS [MPa]
K_5_	9.56 ± 0.81 (A)	251.9 ± 8.9 (A)	8.89 ± 0.71 (A)	13.53 ± 0.95 (A)
(KG_2_)_S_	11.98 ± 0.95 (B)	208.5 ± 12.5 (B)	4.47 ± 0.21 (B)	13.95 ± 0.65 (A)
(G_2_K)_S_	14.98 ± 1.62 (C)	275.7 ± 14.0 (C)	5.88 ± 0.37 (C)	15.07 ± 0.96 (AB)
G_8_	19.79 ± 0.06 (D)	315.8 ± 28.5 (CD)	4.11 ± 0.97 (B)	15.67 ± 1.60 (B)

The A, B, C and D letters in parentheses represent different families (for each column) according to ANOVA (*p* < 0.05).

**Table 3:** Compilation of results from DMA curves shown in Figure 6.

	$E_{g}^{\prime }{}_{\left[-40\;{}^{\circ}C\right]}$ [GPa]	$E_{g}^{\prime }{}_{\left[+40\;{}^{\circ}C\right]}$ [GPa]	C Parameter	T_g_ [°C]	tan δ	A Parameter
Polyurethane	1.96	0.02	--	8.6	0.90	--
K_5_	4.99	0.13	0.35	7.0	0.27	0.74
(KG_2_)_S_	6.50	0.18	0.31	7.7	0.33	0.54
(G_2_K)_S_	12.79	1.14	0.10	8.3	0.41	0.69
G_8_	20.50	2.13	0.08	8.6	0.53	0.59

## Data Availability

All the experimental data presented herein are made available to the corresponding author upon request.
